# Management cessation drives divergent bacterial and fungal community assembly in moso bamboo forests of the central Hunan hilly region, China

**DOI:** 10.3389/fmicb.2026.1880979

**Published:** 2026-08-31

**Authors:** Yong Meng, Meiqun Li, Minggao Chen, Wende Yan, Wensheng Ai, Ming Yang, Chao Peng

**Affiliations:** 1 Faculty of Forestry, Central South University of Forestry and Technology, Changsha City, Hunan, China; 2 State Key Laboratory of Woody Oil Resources Utilization, Hunan Academy of Forestry, Changsha, Hunan, China

**Keywords:** abandonment duration, community assembly, deterministic processes, environmental filtering, management cessation, moso bamboo, stochastic processes

## Abstract

Management cessation and subsequent abandonment alter soil resource availability and chemical conditions, yet how bacterial and fungal communities differ in their responses—in terms of diversity, community structure, and assembly processes—remains poorly resolved in moso bamboo forests. We sampled 27 composite soil samples from three soil depths (0–10, 10–20, and 20–30 cm) across three management regimes, including extensive management (EM), 10-year abandonment (AM10), and 20-year abandonment (AM20), in moso bamboo (Phyllostachys edulis) forests at Taohuajiang State Forest Farm, Taojiang County, Yiyang City, Hunan Province, China. 16S rRNA and ITS amplicon sequencing were combined with measurements of soil physicochemical properties and enzyme activities. Management cessation and subsequent abandonment were associated with distinct soil environmental gradients, especially in total *K*, pH, and ammonium nitrogen. Bacterial Shannon diversity declined monotonically with abandonment duration (EM > AM10 > AM20), while fungal diversity exhibited a significant treatment × depth interaction (*F* = 8.80, BH-adjusted *P* < 0.001), indicating that soil depth modulated fungal responses to abandonment. PERMANOVA explained 77.0% of bacterial community variation but only 34.4% of fungal variation. Assembly analyses revealed that bacterial communities shifted from dispersal limitation (91.7% in EM) toward homogeneous selection (52.8% in AM20), whereas fungi were predominantly governed by stochastic processes (drift or undominated, 75.2%). Bacterial and fungal communities thus showed divergent ecological responses along the abandonment gradient: bacteria exhibited stronger deterministic assembly associated with soil environmental gradients, whereas fungi retained greater stochasticity and depth dependence. These findings clarify the belowground ecological consequences of management cessation in moso bamboo forests of the central Hunan hilly region and highlight the need to consider both bacterial and fungal communities when assessing the ecological effects of bamboo forest abandonment.

## 1 Introduction

Management cessation and subsequent abandonment alter soil resource availability and chemical conditions, yet their consequences for bacterial vs. fungal community assembly processes remain poorly resolved, particularly in moso bamboo forests. Moso bamboo (Phyllostachys edulis) forests in subtropical China have experienced transitions from active or extensive management to progressive abandonment in recent decades. The cessation of harvesting and understory clearing may modify litter input quantity and quality, understory development, rhizosphere processes, and root carbon inputs, which in turn reshape soil pH, nutrient availability, and extracellular enzyme activities, creating divergent niches for bacterial and fungal communities. However, the mechanistic basis by which abandonment-related environmental reorganization after management cessation differentially influences bacterial vs. fungal communities—including their assembly processes—remains insufficiently understood.

Soil microorganisms are key drivers of organic matter decomposition, nutrient mineralization, and humus formation, and are therefore closely linked to bamboo forest ecosystem functioning (Bardgett and van der Putten, 2014; Delgado-Baquerizo et al., 2020). Previous studies have shown that bamboo-associated soil microbial communities are sensitive to land-use change, bamboo expansion, bamboo variety, soil compartment, and management practices. For example, Zhang H. et al. (2022) reported that land-use-driven changes in soil labile carbon were associated with shifts in microbial community composition and function. Zhang X. et al. (2022) further reported that different moso bamboo management practices altered soil microbial functional characteristics related to biomass decomposition. Bamboo invasion has also been shown to affect soil microbial composition, diversity, and abundance in adjacent planted forests (Sun et al., 2023), while bamboo variety and soil compartment can shape fungal community diversity, network structure, and predicted functional guilds (Guo et al., 2023). A recent review further summarized that microbial diversity, community composition, and potential functions in bamboo ecosystems have been increasingly characterized (Wang et al., 2025).

However, the central gap is not whether microbial communities differ among bamboo forest types or management regimes, but whether bacterial and fungal communities are assembled through similar or different ecological processes after management cessation. Most existing studies have focused mainly on diversity patterns, taxonomic composition, environmental correlations, functional genes, or predicted functions. Less attention has been given to the balance between deterministic processes, such as environmental filtering, and stochastic processes, such as dispersal limitation and drift, especially when bacterial and fungal communities are compared across soil depths within the same abandonment-duration gradient (Stegen et al., 2012; Zhou and Ning, 2017). Therefore, explicitly linking compositional shifts to environmental gradients while partitioning community assembly processes provides a process-based extension beyond previous diversity-, composition-, and function-focused studies in bamboo ecosystems.

To address these gaps, we integrated soil physicochemical data, extracellular enzyme activities, and amplicon sequencing data (16S rRNA and ITS) across three management regimes—extensive management, 10-year abandonment, and 20-year abandonment—in moso bamboo forests at Taohuajiang State Forest Farm in the central Hunan hilly region, China. We hypothesized that soil environmental shifts following management cessation would be associated with divergent community responses: bacteria would exhibit stronger and more deterministic responses to abandonment-related environmental gradients, whereas fungi would show greater depth dependence and higher proportions of stochastic assembly, reflecting their contrasting ecological traits. By focusing on assembly processes rather than only diversity and taxonomic composition, this study provides a simultaneous comparison of bacterial and fungal community assembly across soil depths and abandonment duration in moso bamboo forests of the central Hunan hilly region.

## 2 Materials and methods

### 2.1 Study site and experimental design

The study was conducted at Taohuajiang State Forest Farm, Taojiang County, Yiyang City, Hunan Province, China (112°04′16″-112°06′59″ E, 28°18′25″-28°20′13″ N), which is located in the central Hunan hilly region and has a subtropical monsoon climate. The selected plots were developed on yellow-brown earths typical of the central Hunan hilly region. Common understory species include *Euscaphis japonica, Kadsura longipedunculata, Aralia chinensis, Sabia japonica, Veronicastrum stenostachyum, Phyllanthus glaucus, Millettia reticulata*, and *Woodwardia japonica*.

Three management regimes were selected according to the historical management records of Taohuajiang State Forest Farm and field confirmation by workers familiar with the long-term management history of the bamboo stands: extensive management (EM), 10-year abandonment (AM10), and 20-year abandonment (AM20). EM stands were selectively harvested every 2–3 years, and understory shrubs and weeds were cleared every 3–5 years. In this study, abandonment refers to the cessation of direct human management activities, including selective harvesting and understory clearing, followed by natural stand development. In AM10 and AM20 stands, these direct management activities had ceased for approximately 10 and 20 years, respectively. AM10 and AM20 were selected to represent two contrasting durations after management cessation: AM10 reflects an intermediate abandonment stage with ongoing understory recovery and litter accumulation, whereas AM20 reflects a longer-term abandonment stage in which stand structure and soil microbial habitats may have moved further toward natural development. Three independent 20 m × 20 m plots per regime served as biological replicates.

AM10 and AM20 plots were approximately 300–500 m apart, whereas EM plots were approximately 3–5 km from the abandonment plots because of the historical distribution of management units within the forest farm. This spatial arrangement may introduce geographic confounding between treatments because management history and spatial location were not fully independent. To partially mitigate non-independence arising from the split-plot structure, PERMANOVA permutations were stratified at the plot level.

Within each 20 m × 20 m plot, five soil cores were collected along an S-shaped transect at three depth intervals (0–10, 10–20, and 20–30 cm) and homogenized into one composite soil sample per depth, yielding 27 composite samples in total (3 regimes × 3 replicate plots × 3 depths).

### 2.2 Soil physicochemical properties and enzyme activity measurements

All 27 samples were analyzed for total nitrogen, total phosphorus, total potassium (K), available nitrogen, phosphorus and potassium, soil organic matter, pH, ammonium nitrogen (${\text{NH}}_{4}^{+}$-N), nitrate nitrogen (${\text{NO}}_{3}^{-}$-N), humus fractions, and six enzyme activities: urease, acid phosphatase, cellulase, β-glucosidase, peroxidase, and polyphenol oxidase. Initial screening used linear mixed-effects models (LMMs); variables with significant treatment effects (Benjamini–Hochberg [BH]-adjusted *P* < 0.05) were retained. Collinear variables (|*r*| > 0.7) were removed, yielding six core variables: total *K*, pH, organic matter, ammonium nitrogen, cellulase activity, and polyphenol oxidase activity (Supplementary Table S1). Soil pH was measured potentiometrically in a soil-water suspension. Soil organic matter was determined using the potassium dichromate oxidation method. Total *K* was measured by microwave digestion followed by ICP-MS. Ammonium nitrogen was extracted with KCl and determined using the indophenol blue colorimetric method. Cellulase and polyphenol oxidase activities were measured using commercial assay kits following the manufacturer's protocols.

### 2.3 Amplicon sequencing and bioinformatic processing

Bacterial communities were profiled using 16S rRNA gene V3–V4 amplicons and fungal communities using ITS1 amplicons, sequenced on the MGI DNBSEQ-G99 platform (PE300). Raw sequences were processed through the DADA2 pipeline (Callahan et al., 2016), encompassing quality filtering, denoising, paired-end merging, chimera removal, and amplicon sequence variant (ASV) table construction. Taxonomy was assigned against SILVA SSU r138.2 (16S) and UNITE (ITS). All diversity and community analyses were performed at the ASV level. For visualization, bacterial communities are displayed at phylum and genus levels; fungal communities at the phylum level; low-abundance taxa are grouped as “Other.”

### 2.4 Community diversity and structure analyses

ASV tables were rarefied to the minimum sequencing depth across all retained samples after quality filtering before α- and β-diversity analyses. DESeq2 differential abundance analyses used its built-in normalization on raw counts. β-diversity was calculated as Bray–Curtis dissimilarity and visualized by principal coordinates analysis (PCoA). Community composition differences were tested by PERMANOVA (999 permutations; vegan::adonis2), with permutations restricted at the plot level to account for split-plot non-independence. Homogeneity of within-group dispersions was confirmed by betadisper before interpreting PERMANOVA results. Environmental drivers were examined by distance-based redundancy analysis (dbRDA; Legendre and Anderson, 1999), after standardizing environmental variables and controlling collinearity by pairwise correlation (|r| > 0.7) and variance inflation factor (VIF > 10). Envfit was used to evaluate associations between environmental variables and ordination axes. Relationships between representative taxa and environmental gradients (total *K*, pH, ${\text{NH}}_{4}^{+}$-N, cellulase activity) were explored with linear models and generalized additive models (GAMs). All analyses used R v4.3.3 (vegan, lme4, phyloseq, ggplot2).

### 2.5 Linear mixed-effects models and response variable screening

Soil variables, enzyme activities, and α-diversity indices were analyzed using LMMs (response ~ treatment × depth + (1 | plot)) with treatment and depth as fixed effects and plot as a random effect. *P*-values were BH-corrected. Results are summarized in Supplementary Table S1.

### 2.6 Differential abundance, *post hoc* comparisons, and environmental associations

Representative differentially abundant taxa were identified by DESeq2 pairwise comparisons, interpreted conservatively given the compositional nature of amplicon data. Robustness was assessed using DESeq2, ANCOM-BC, and ALDEx2. Taxa were defined as robust differential taxa only when they were detected as significant by at least two of the three methods, with BH-adjusted *P* < 0.05 in each corresponding analysis. Taxa detected by only one method were treated as provisional signals and interpreted conservatively. For representative taxa, *post hoc* pairwise LMM comparisons clarified treatment-level differences. Spearman correlations between taxa and environmental variables were BH-corrected.

### 2.7 Community assembly processes and variance partitioning

The relative contributions of deterministic and stochastic assembly were quantified using the β-nearest taxon index (βNTI) and RC_Bray_ framework (Stegen et al., 2012). A maximum-likelihood phylogenetic tree was built using FastTree v2.1.11 (GTR+Gamma model) on MAFFT v7.505-aligned 16S ASV sequences. The βNTI null model used 999 randomizations. Classification followed Stegen et al. (2012): βNTI < −2, homogeneous selection; βNTI > +2, variable selection; |βNTI| ≤ 2 with RC_Bray_ > 0.95, dispersal limitation; RC_Bray_ < −0.95, homogenizing dispersal; remainder, drift or undominated. Variance partitioning (varpart) decomposed community variation into pure and shared effects of three predictor sets: management treatment, soil depth, and environmental variables (after collinearity screening). Adjusted *R*^2^ values are reported throughout.

### 2.8 Supportive functional prediction analyses

Bacterial functional potential was inferred from 16S data using PICRUSt2 v2.5.1 (sequence placement, hidden-state prediction, MetaCyc pathway reconstruction) (Douglas et al., 2020). Only functions relevant to carbon decomposition, nitrogen cycling, and phosphorus transformation were retained. Fungal ecological guilds were annotated using FUNGuild (Nguyen et al., 2016) and FungalTraits (Põlme et al., 2020); only saprotrophic guilds were retained. These inferences represent predicted functional potential rather than direct measurements of gene abundances, transcript activity, enzyme kinetics, or ecosystem process rates. Because PICRUSt2 depends on phylogenetic placement and reference genome coverage, and fungal guild annotation depends on incomplete trait databases, these results were treated only as supportive and hypothesis-generating evidence.

### 2.9 Cross-kingdom association network analysis

Treatment-specific bacterial-fungal association networks were constructed using FlashWeave (Tackmann et al., 2019) to explore potential changes in microbial association patterns across management treatments along the abandonment gradient. Bacterial and fungal abundance tables were combined after taxonomic filtering, and separate networks were inferred for EM, AM10, and AM20. Network nodes represent bacterial or fungal taxa, and edges represent statistical associations inferred by FlashWeave. Only connected taxa were displayed for visualization clarity, whereas network topological parameters were calculated from the full treatment-specific networks. The number of connected taxa, edge number, network density, average degree, clustering coefficient, modularity, and the number and proportion of bacterial-fungal edges were summarized for each treatment. Because network edges represent statistical associations rather than direct biological interactions, the network results were interpreted as exploratory evidence of cross-kingdom association patterns.

## 3 Results

### 3.1 Soil environmental conditions varied along the abandonment gradient

**Figure 1:**
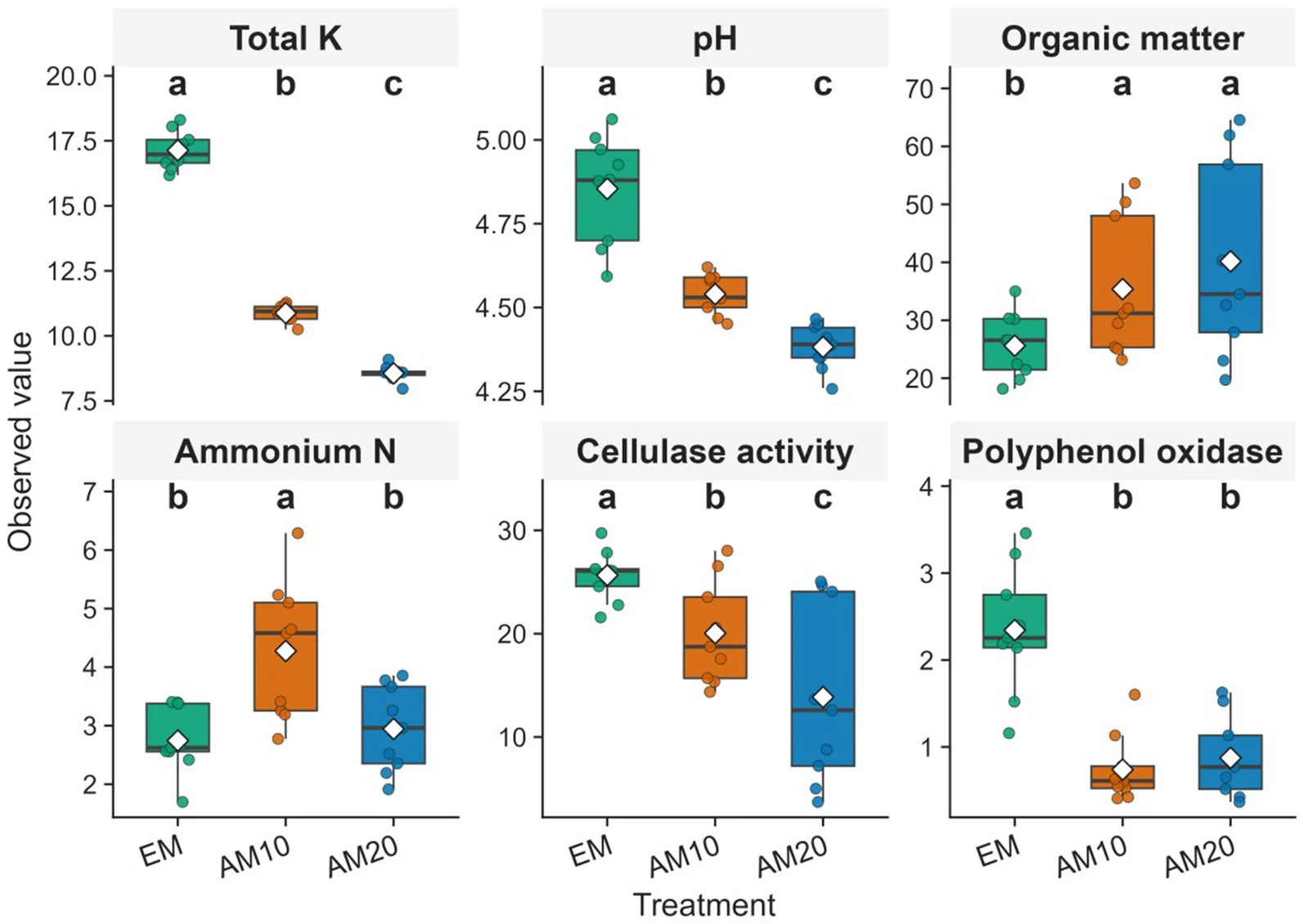
Variation in key soil environmental variables across management treatments. The six core variables are shown: total *K*, pH, organic matter, ammonium nitrogen, cellulase activity, and polyphenol oxidase activity. Boxplots show within-treatment distributions; central lines indicate medians; white diamonds represent means; points represent individual samples. Different lowercase letters indicate significant differences among treatments based on *post hoc* pairwise comparisons from linear mixed-effects models with BH correction (*P* < 0.05).

All six core environmental variables responded significantly to management treatment (LMM Treatment main effect—total *K*: *F* = 637.2; pH: *F* = 330.2; organic matter: *F* = 48.1; ammonium nitrogen: *F* = 22.7; cellulase activity: *F* = 65.9; polyphenol oxidase activity: *F* = 24.0; all BH-adjusted *P* < 0.001; Figure 1; Supplementary Table S1). Total *K*, pH, and cellulase activity declined monotonically with abandonment duration (EM > AM10 > AM20). Organic matter was higher in AM10 and AM20 than in EM. Ammonium nitrogen was highest in AM10. Polyphenol oxidase activity was significantly higher in EM than in both abandonment treatments.

### 3.2 Contrasting diversity responses of bacterial and fungal communities

**Figure 2:**
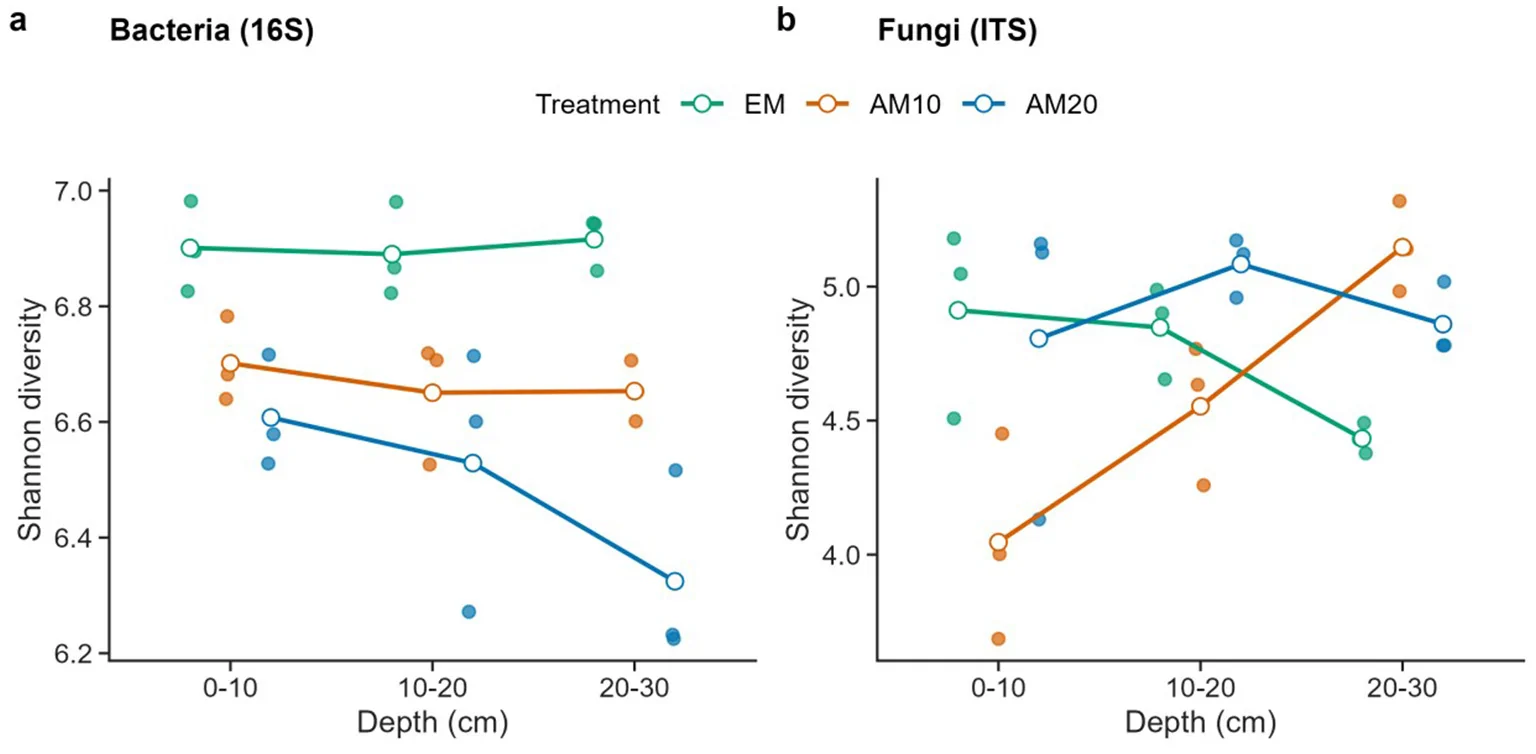
Shannon diversity of bacterial and fungal communities across management treatments and soil depths. **(a)** 16S bacterial community; **(b)** ITS fungal community. Colored points represent individual samples; open circles and connecting lines show mean trends across soil depths within each treatment. Colors denote treatments; the x-axis represents soil depth. This figure illustrates the management main effect and its interaction with soil depth.

Bacterial Shannon diversity responded significantly to management treatment (*F* = 30.88, BH-adjusted *P* < 0.001), declining monotonically with abandonment duration (EM > AM10 > AM20; Figure 2a). In contrast, the treatment × depth interaction was the dominant statistical signal for fungal ITS Shannon diversity (*F* = 8.80, BH-adjusted *P* < 0.001), although the treatment main effect was also significant (*F* = 4.38, BH-adjusted *P* = 0.032; Figure 2b). Specifically, AM10 exhibited higher fungal Shannon diversity in surface soils (0–10 cm) relative to EM and AM20, whereas this pattern was attenuated in deeper layers (20–30 cm). Thus, bacterial Shannon diversity showed a treatment main effect, whereas fungal Shannon diversity showed significant treatment and treatment × depth effects.

### 3.3 Community structure differentiation and environmental drivers

**Figure 3:**
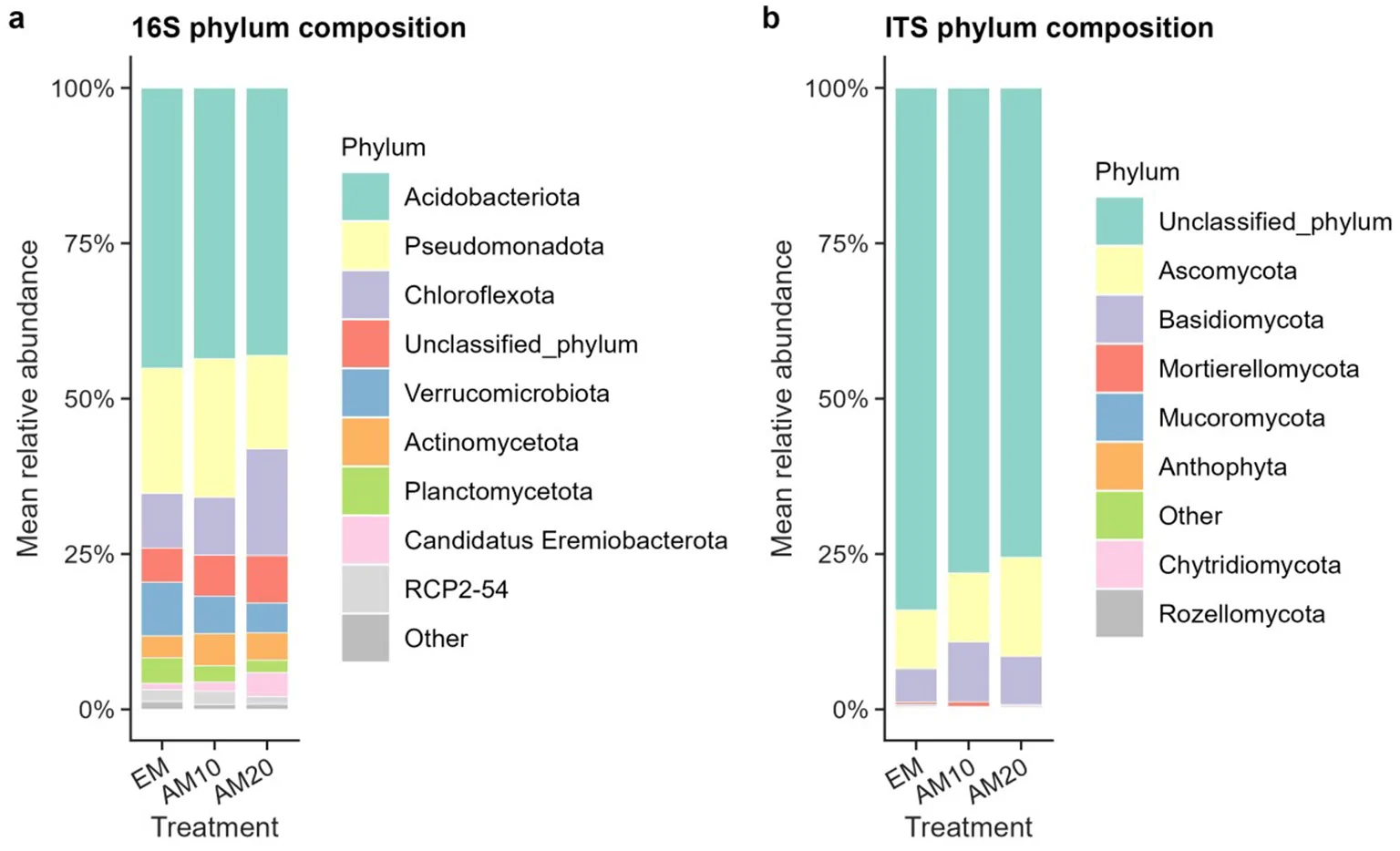
Phylum-level community composition of bacterial and fungal communities across management treatments. **(a)** 16S bacterial community; **(b)** ITS fungal community. Each bar represents the mean relative abundance within a treatment, normalized to 100%. Only dominant phyla are displayed; low-abundance taxa are grouped as “Other”.

**Figure 4:**
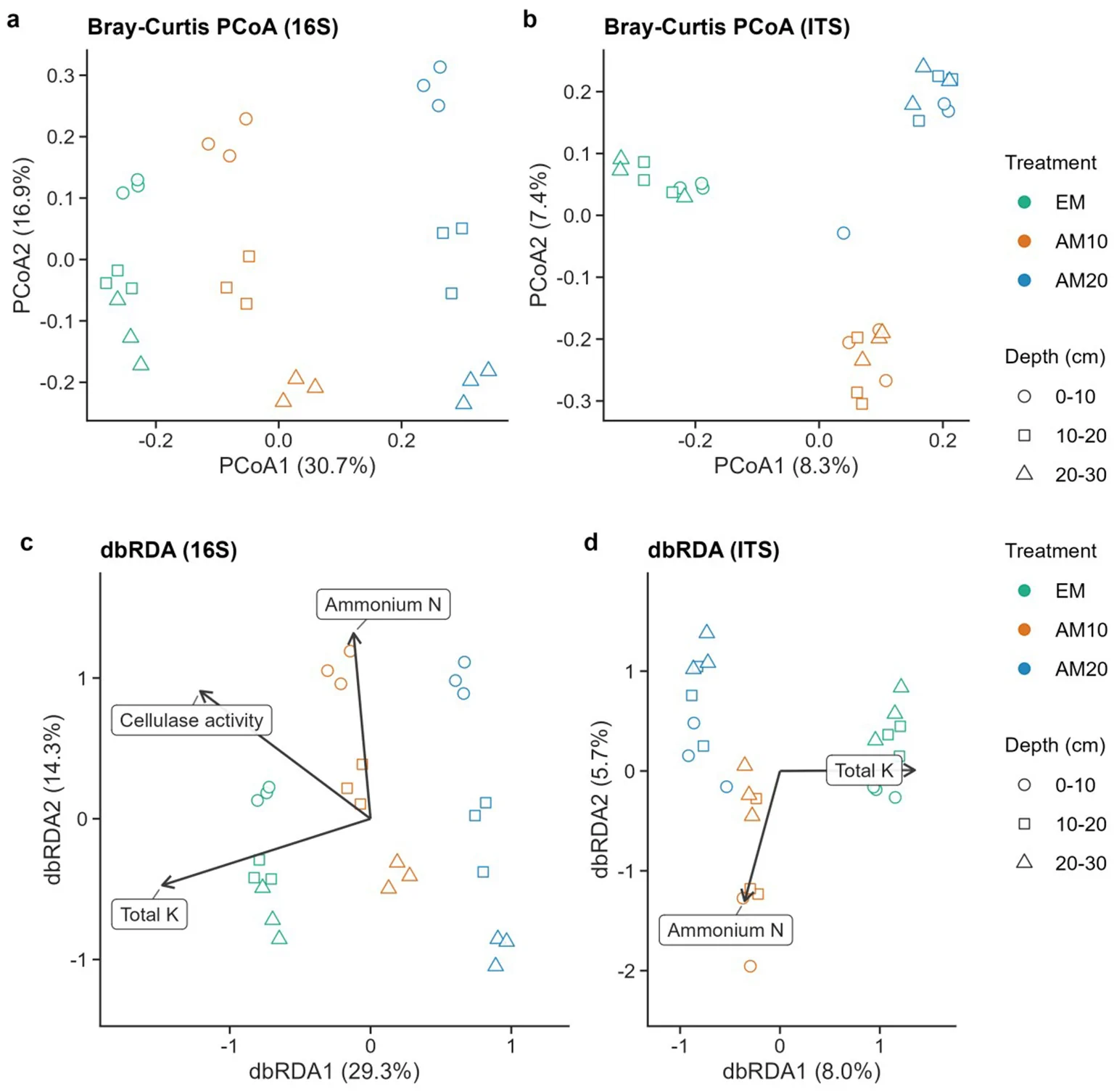
Ordination and constrained ordination of bacterial and fungal communities based on Bray–Curtis distances. **(a)** 16S PCoA; **(b)** ITS PCoA; **(c)** 16S dbRDA; **(d)** ITS dbRDA. Colors represent management treatments; point shapes indicate soil depth. Arrows in dbRDA panels show selected significant environmental variables and their direction and strength of association with community variation. PCoA illustrates community separation; dbRDA shows how treatment-associated environmental factors explain community variation.

Bacterial communities were dominated by Acidobacteriota, Pseudomonadota, and Chloroflexota, with Chloroflexota notably more abundant in AM20. Fungal communities showed a high proportion of unclassified phyla, with Ascomycota and Basidiomycota exhibiting treatment-associated shifts (Figure 3). PERMANOVA detected significant treatment effects on both bacterial and fungal communities, with higher explanatory power for 16S than ITS data (16S: *F* = 10.04, *R*^2^ = 0.770, *P* = 0.003; ITS: *F* = 1.58, *R*^2^ = 0.344, *P* = 0.004; Figures 4a, b). dbRDA showed that bacterial community variation was associated mainly with total *K*, ammonium nitrogen, and cellulase activity, whereas fungal community variation was associated mainly with total *K* and ammonium nitrogen (Figures 4c, d). Depth-resolved phylum-level composition patterns across treatments and soil depths are provided in Supplementary Figure S1.

### 3.4 Community assembly processes and variance partitioning

**Figure 5:**
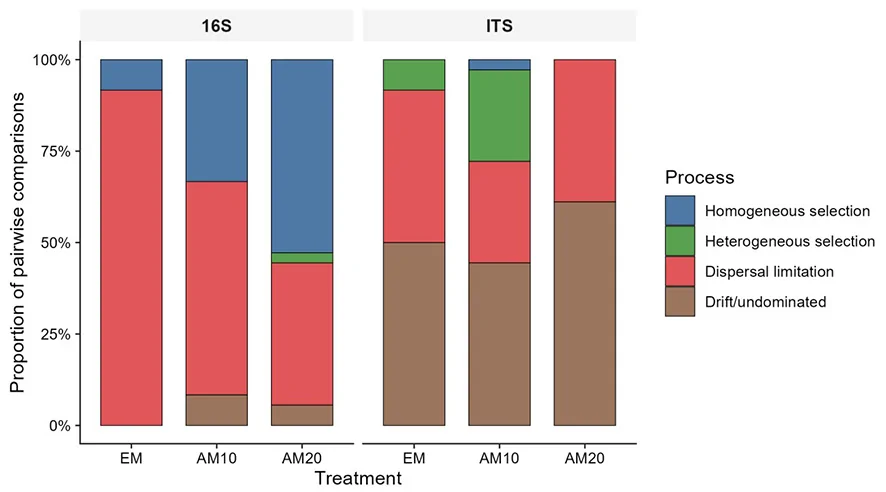
Community assembly processes of bacterial and fungal communities across management treatments. Left: 16S bacterial community; right: ITS fungal community. Stacked bar charts show the proportions of pairwise sample comparisons classified as homogeneous selection, variable selection, dispersal limitation, homogenizing dispersal, or drift/undominated processes, for each treatment and overall.

βNTI and RCBray analyses showed different assembly profiles between bacterial and fungal communities (Figure 5). Overall, bacterial community turnover consisted of homogeneous selection (51.9%), dispersal limitation (20.5%), homogenizing dispersal (15.1%), and drift or undominated processes (12.5%). In EM, bacterial communities were dominated by dispersal limitation (91.7%). In AM10, dispersal limitation and homogeneous selection accounted for 58.3% and 33.3%, respectively. In AM20, homogeneous selection accounted for 52.8%. Fungal communities were dominated by drift or undominated processes overall (75.2%), with dispersal limitation accounting for 11.1%. Drift or undominated processes accounted for 50.0%, 44.4%, and 61.1% in EM, AM10, and AM20, respectively.

**Figure 6:**
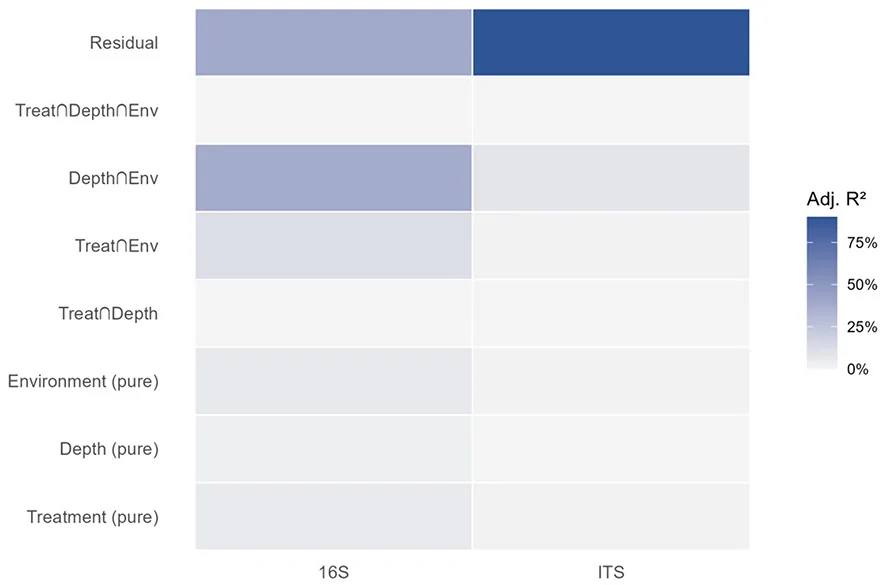
Variance partitioning of bacterial and fungal community variation. The 16S bacterial community is shown in the left column, and the ITS fungal community is shown in the right column. The figure shows pure and shared effects of management treatment, soil depth, and environmental variables. Values are adjusted *R*^2^ (Adj. *R*^2^); negative values indicate contributions below random expectation.

Variance partitioning showed that the pure environmental fraction was the largest individual contributor to bacterial community variation (adjusted *R*^2^ ≈ 0.064), followed by pure treatment (≈0.050) and pure depth (≈0.028) effects (Figure 6). For fungal communities, pure treatment and environmental fractions were lower (0.008–0.012), and the pure depth fraction was approximately zero.

### 3.5 Representative differentially abundant taxa and robustness assessment

**Figure 7:**
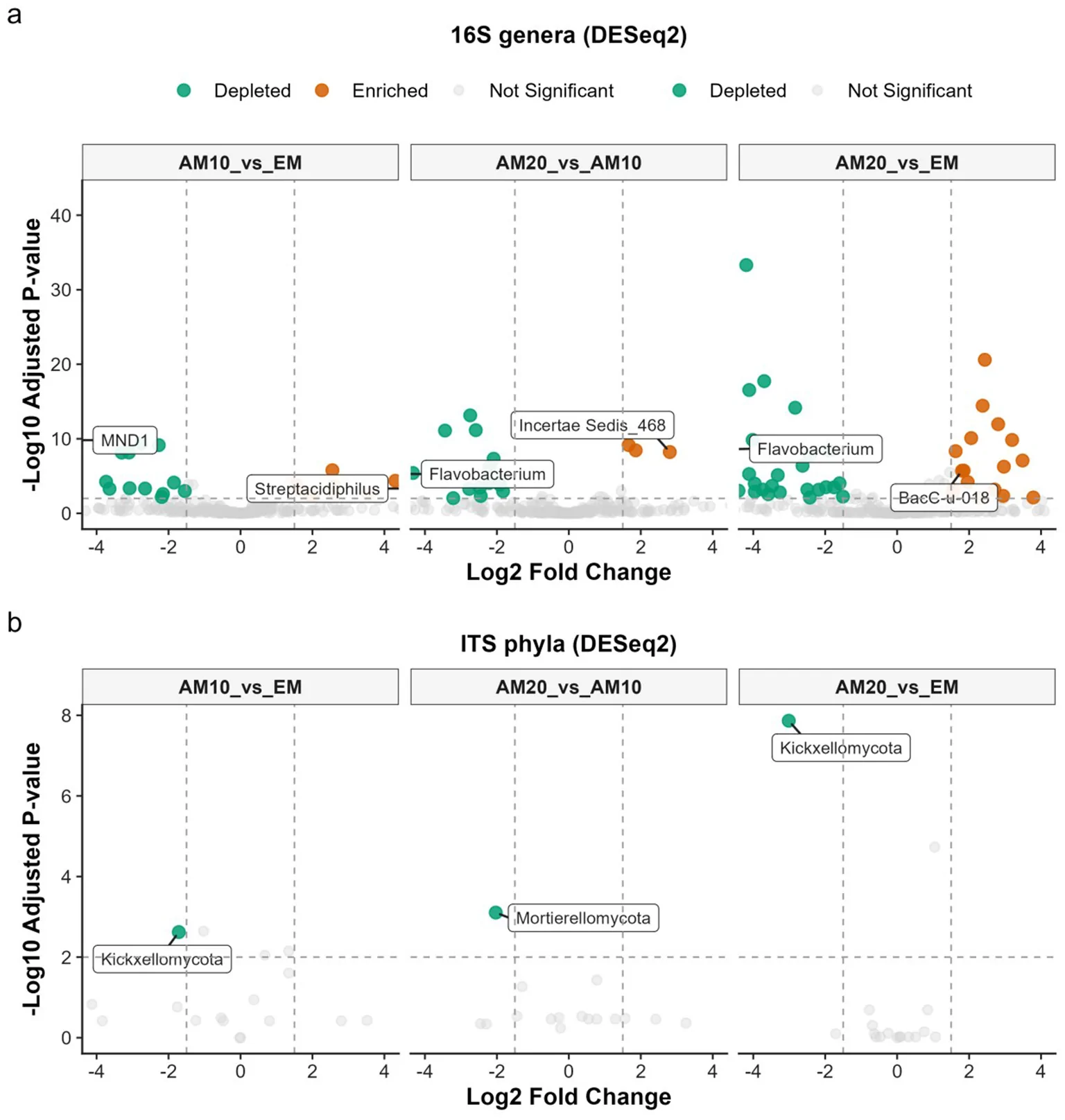
Volcano plots of representative differentially abundant taxa from pairwise DESeq2 comparisons. **(a)** 16S at the genus level; **(b)** ITS at the phylum level. Each column corresponds to a treatment contrast. The x-axis shows log_2_ fold change; the y-axis shows –log_10_ adjusted *P*-values. Orange points: enriched taxa; green points: depleted taxa; gray points: non-significant taxa. Only representative taxa with clear ecological relevance are labeled.

**Figure 8:**
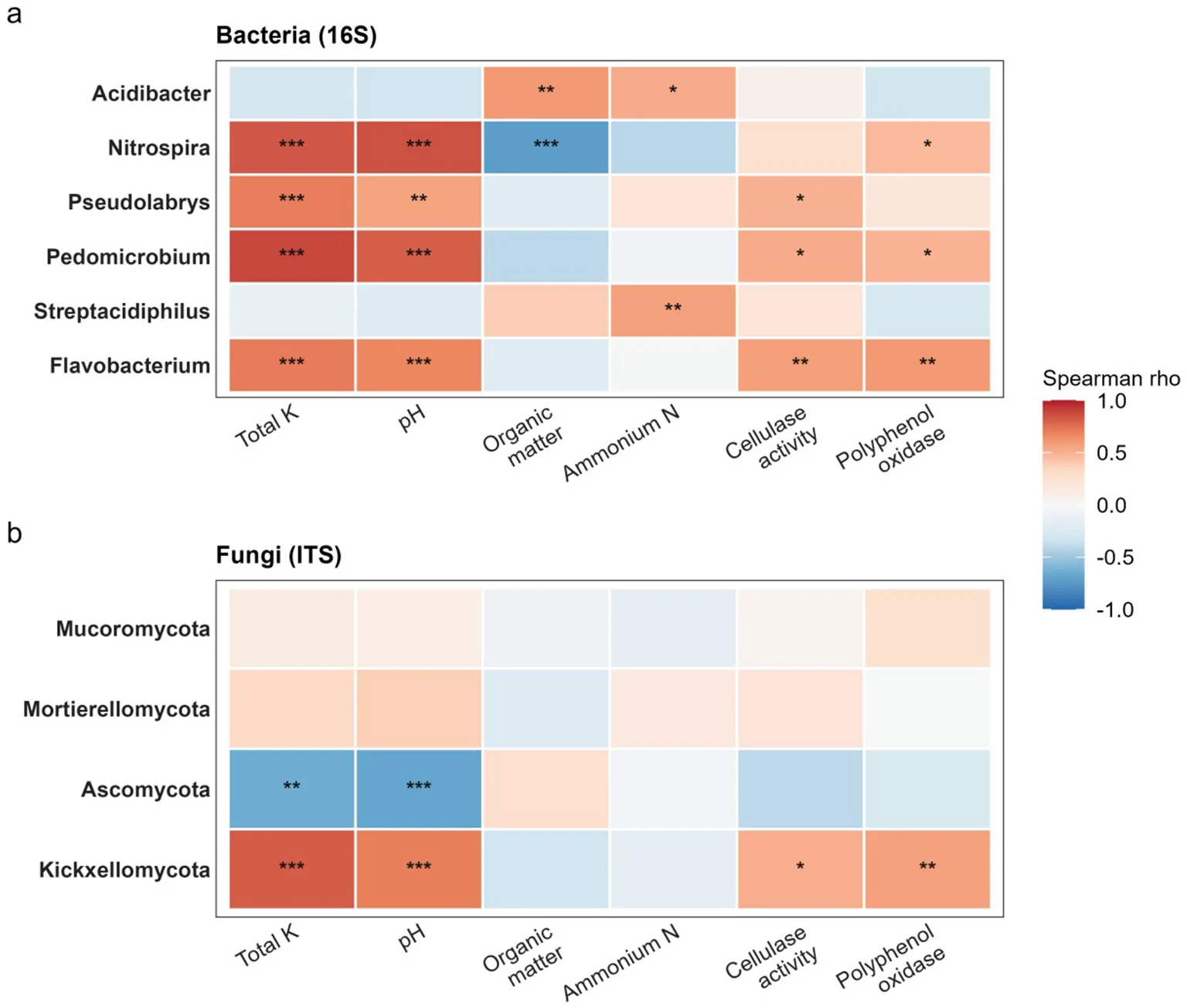
Correlations between key microbial taxa and soil environmental variables. **(a)** Key bacterial genera; **(b)** key fungal phyla. Colors indicate the direction and strength of Spearman correlations (red: positive; blue: negative). Asterisks denote significance after BH correction: *P < 0.05; **P < 0.01; ***P < 0.001.

**Figure 9:**
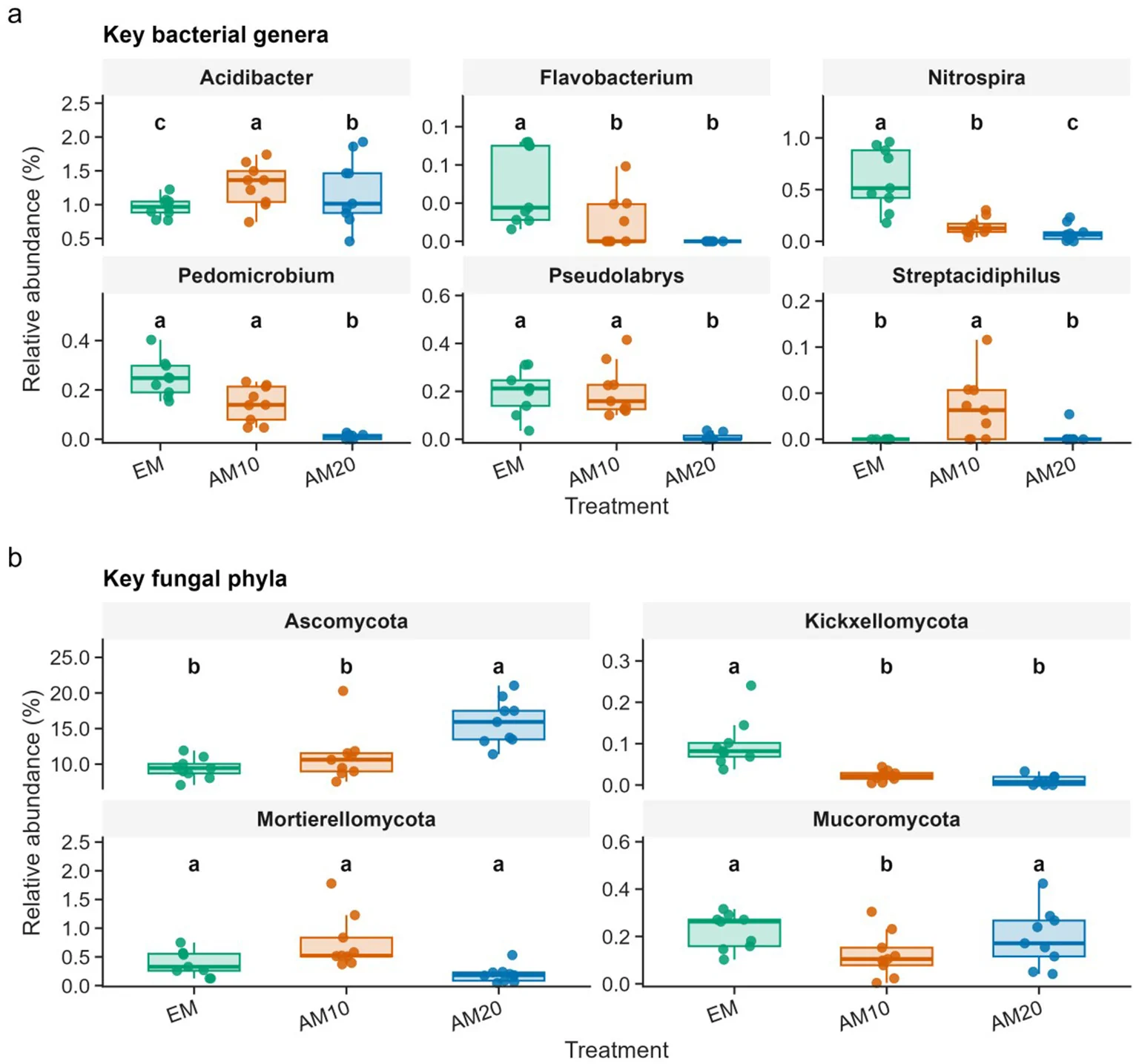
Relative abundance of key microbial taxa across management treatments. **(a)** Key bacterial genera; **(b)** key fungal phyla. Boxplots show within-treatment distributions; points represent individual samples. Different lowercase letters indicate significant differences among treatments (BH-adjusted *P* < 0.05).

DESeq2 identified representative differentially abundant taxa across management contrasts (Figure 7). In bacterial communities: MND1 decreased and *Streptacidiphilus* increased in AM10 relative to EM; *Flavobacterium* decreased in AM20 relative to both EM and AM10. In fungal communities, *Kickxellomycota* decreased in AM10 and AM20 relative to EM; *Mortierellomycota* decreased in AM20 relative to AM10. Sensitivity analyses (ANCOM-BC and ALDEx2) confirmed robust signals for MND1, *Flavobacterium*, and *Mortierellomycota*, each supported by at least two methods; remaining taxa were treated as provisional signals and interpreted conservatively. Correlation analysis showed that several representative taxa were associated with core environmental gradients, particularly total *K* and pH (Figure 8), and their relative abundance patterns differed among treatments (Figure 9). Depth-specific patterns of these representative taxa are summarized in Supplementary Figure S2, and additional bacterial and fungal taxon-environment response curves are provided in Supplementary Figures S5 and S6.

### 3.6 Cross-kingdom association networks varied across management treatments

**Figure 10:**
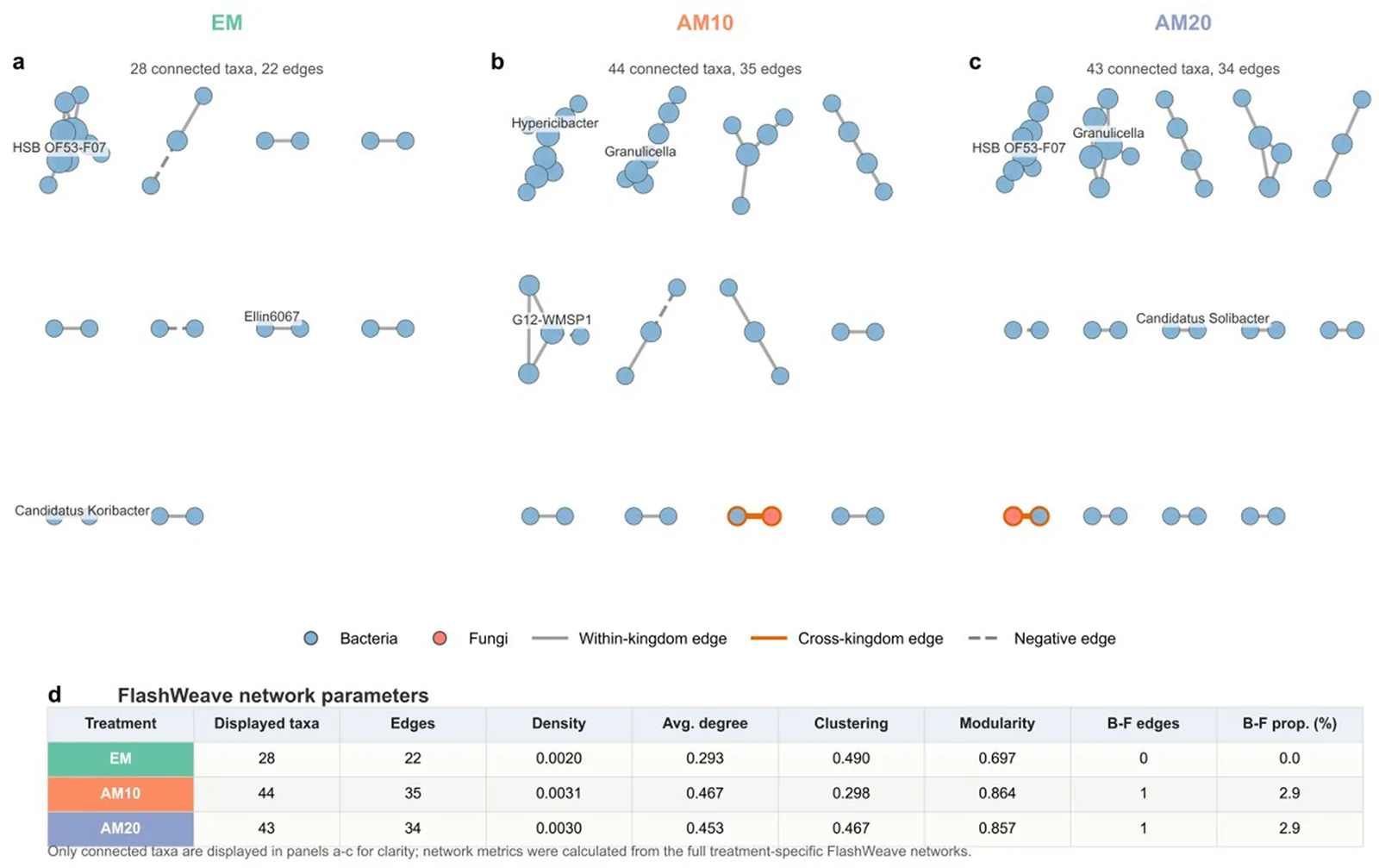
Treatment-specific bacterial-fungal association networks inferred by FlashWeave. Panels **(a–c)** show the treatment-specific networks for EM, AM10, and AM20, respectively. Only connected taxa are displayed for visualization clarity. Blue nodes represent bacteria, and red nodes represent fungi; node size is proportional to node degree. Gray solid lines indicate within-kingdom associations, orange solid lines indicate bacterial-fungal associations, and gray dashed lines indicate negative associations. Panel **(d)** summarizes the main network topological parameters, including the number of displayed taxa, edge number, density, average degree, clustering coefficient, modularity, and the number and proportion of bacterial-fungal edges. Network metrics were calculated from the full treatment-specific FlashWeave networks. Edges represent statistical associations rather than direct biological interactions.

Treatment-specific FlashWeave networks showed that bacterial-fungal association patterns differed among management treatments along the abandonment gradient (Figure 10). EM contained fewer connected taxa and edges than the two abandonment treatments, with 28 connected taxa and 22 edges. AM10 and AM20 showed more connected taxa and edges, with 44 connected taxa and 35 edges in AM10 and 43 connected taxa and 34 edges in AM20. Network density and average degree were also slightly higher in AM10 and AM20 than in EM. Cross-kingdom bacterial-fungal edges were rare across all treatments: no bacterial-fungal edge was detected in EM, whereas AM10 and AM20 each contained one bacterial-fungal edge, accounting for 2.9% of total displayed edges.

## 4 Discussion

### 4.1 Divergent bacterial and fungal assembly processes after management cessation

The main contribution of this study was the comparison of bacterial and fungal community assembly processes along an abandonment-duration gradient. Previous studies in bamboo ecosystems mainly described shifts in microbial diversity, community composition, environmental associations, or predicted functions. In contrast, the present study showed that bacterial and fungal communities differed not only in composition but also in the ecological processes underlying community turnover. Bacterial communities shifted from dispersal limitation in EM toward a higher proportion of homogeneous selection in AM20, whereas fungal communities remained dominated by stochastic processes. This divergence may indicate that bacteria and fungi followed different assembly pathways after management cessation.

The shift from dispersal limitation toward homogeneous selection in bacterial communities along the EM–AM10–AM20 gradient may indicate that deterministic assembly processes became more prominent with increasing abandonment duration. This pattern was consistent with stronger environmental filtering under long-term abandonment, but it should not be interpreted as direct evidence that the measured soil variables became more homogeneous within AM20. The measured environmental gradients, including total *K*, pH, ammonium nitrogen, and enzyme activities, may represent plausible filtering axes for bacterial community differentiation, whereas within-treatment environmental heterogeneity was not explicitly tested in the present study. Variance partitioning also supported an association between bacterial community variation and abandonment-related environmental reorganization, particularly through the shared explanatory fraction of management and environmental variables. In contrast, the predominance of stochastic processes in fungal communities, together with their lower explained variation, may indicate that fungal reorganization was affected by microhabitat heterogeneity, substrate history, and colonization contingency not fully captured by the measured environmental variables.

An alternative, non-exclusive explanation is successional convergence after management cessation. In EM stands, periodic harvesting and understory clearing may repeatedly disturb local microhabitats and maintain stronger spatial contingency in bacterial colonization, thereby contributing to dispersal-limited community turnover. After long-term abandonment, the cessation of repeated disturbance may allow bacterial communities to converge toward more similar later-stage successional states. In these states, deterministic processes such as competition, substrate specialization, and niche filtering may become more pronounced, leading to a higher proportion of homogeneous selection. Therefore, the observed EM-to-AM20 transition in bacterial assembly should be interpreted as stronger deterministic structuring associated with long-term abandonment, potentially reflecting both abandonment-related environmental filtering and successional convergence, rather than measured environmental homogenization alone. However, given the limited number of replicate plots, βNTI-based assembly estimates should be interpreted cautiously, and larger-scale sampling is needed to confirm this pattern.

### 4.2 Management cessation is associated with soil environmental reorganization, with important spatial caveats

Management cessation and subsequent abandonment were associated with systematic shifts in soil environmental conditions, particularly in total *K*, pH, ammonium nitrogen, and decomposition-related enzyme activities. These findings were consistent with previous studies reporting that water and fertilizer management (Li et al., 2024), nitrogen addition (Cao et al., 2024), litter manipulation (Ge et al., 2022), and bamboo range expansion (Wu et al., 2024) altered soil pH, nutrient status, enzyme activity profiles, or microbial communities in moso bamboo forests. Total *K* declined monotonically with abandonment, which may reflect changes in nutrient cycling, litter quality, root uptake, and leaching processes after management cessation. The decline in pH may be associated with organic acid release during litter decomposition, nitrification-derived proton production, and altered root exudate inputs. These patterns indicate that soil chemical responses after bamboo forest abandonment are likely context dependent and may vary with local soil background, management history, and substrate conditions. These environmental changes are consistent with the broader principle that changes in forest management history can alter soil resource availability and physicochemical conditions, thereby indirectly modifying microbial colonization and competitive dynamics (Bardgett and van der Putten, 2014; Delgado-Baquerizo et al., 2020).

A major design limitation is that management history and spatial location were not fully independent. AM10 and AM20 plots were approximately 300–500 m apart, whereas EM plots were approximately 3–5 km from the abandonment plots because of the historical distribution of management units within Taohuajiang State Forest Farm. All selected plots were located within the same forest farm in the central Hunan hilly region and represented typical moso bamboo stands developed on yellow-brown earths. This regional consistency supports a broadly comparable environmental background among treatments. However, potential differences in parent material, topographic position, microclimate, or earlier land-use history cannot be fully excluded. Therefore, the observed community differences should be interpreted as management-history-associated patterns within this local forest-farm context, rather than as fully isolated causal effects of management cessation. The high bacterial PERMANOVA *R*^2^ should also be interpreted in light of the limited number of replicate plots, which may inflate between-treatment explanatory power. Future studies should employ paired-plot designs within the same hillslope or watershed and larger sample sizes to more rigorously isolate management effects.

### 4.3 Bacteria and fungi exhibit contrasting response modes across abandonment and depth gradients

The divergent assembly patterns described above were accompanied by contrasting diversity and compositional responses of bacterial and fungal communities along the abandonment gradient. Bacterial communities showed clear treatment main effects in both α- and β-diversity, while fungal communities—although also affected by treatment—displayed more prominent treatment × depth interactions. This pattern may indicate that bacterial communities responded more clearly to abandonment-related soil environmental gradients, whereas fungal communities retained a stronger modulatory role of the soil depth context (de Vries et al., 2018). Such divergence aligns with the contrasting ecological traits of the two kingdoms: bacteria have faster turnover rates and are highly sensitive to changes in pH, ammonium nitrogen, and labile resource availability (Lauber et al., 2009), making them more responsive to abandonment-related environmental gradients. Fungi—particularly saprotrophic guilds—depend more strongly on complex organic substrates and local microhabitat conditions; their distributions are additionally constrained by soil profile structure and substrate accumulation history (Bahram et al., 2018; Mundra et al., 2021). Differences in litter layer thickness across management treatments along the abandonment gradient may generate distinct substrate availability profiles in surface vs. deep soils, producing the treatment × depth interactive patterns observed for fungi. From an assembly perspective, this differential responsiveness underpins the stronger deterministic assembly of bacteria and the higher stochasticity retained by fungi, consistent with theoretical expectations (Zhou and Ning, 2017; Zhao et al., 2019).

### 4.4 Directional taxon turnover along abandonment-related environmental gradients

Key taxa showed directional shifts along total *K*, pH, and ammonium nitrogen gradients, with robust signals corroborated by multiple differential abundance methods. The decline of Flavobacterium with increasing abandonment is ecologically interpretable because this genus is often associated with organic matter degradation and tends to respond sensitively to soil nutrient availability and pH. Its depletion in abandoned plots, where pH and cellulase activity declined, is consistent with the shift in soil chemical conditions and substrate availability along the abandonment gradient. MND1, an uncharacterized lineage within Chloroflexota, has limited documented ecological functions and should therefore be interpreted cautiously. For Mortierellomycota, the lower abundance in AM20 may reflect altered litter quality and substrate availability under long-term abandonment, although this interpretation remains tentative because ITS-based taxonomic resolution and functional inference are limited.

### 4.5 Predicted functional shifts are directionally consistent but require direct verification

PICRUSt2 predictions and fungal guild annotations suggested that predicted functional composition varied across management treatments in directions partly aligned with environmental gradients and taxon turnover. For example, predicted pathways related to carbon transformation and nitrogen cycling showed treatment-associated changes (Supplementary Figures S3, S4, and S7). However, these results represent functional potential inferred from 16S and ITS amplicon data rather than direct measurements of gene abundances, transcript activity, enzyme kinetics, or ecosystem process rates. PICRUSt2 predictions depend on phylogenetic placement and reference genome coverage, whereas fungal guild annotations depend on incomplete fungal trait databases. These limitations may be particularly relevant in moso bamboo forests, where many bacterial and fungal taxa remain poorly characterized.

Functional redundancy further constrains the ecological interpretation of these predictions. Some taxa that changed across management treatments along the abandonment gradient, such as *Flavobacterium, Pedomicrobium*, and related bacterial groups, may share partially overlapping roles in organic matter decomposition or nutrient transformation. If functionally similar taxa compensate for one another, marked compositional shifts may not necessarily translate into proportional changes in ecosystem processes. Future work should therefore combine amplicon sequencing with metagenomics, metatranscriptomics, enzyme kinetics, and direct process-rate measurements to determine whether the observed taxonomic turnover leads to measurable changes in decomposition and nutrient cycling.

### 4.6 Limitations and future directions

Five key limitations should be acknowledged. (1) Spatial confounding: management history and spatial location were not fully independent, and differences in parent material, topography, microclimate, or earlier land-use history cannot be fully excluded. (2) Temporal snapshot: the cross-sectional design precludes direct inference about successional trajectories. Therefore, the inferred successional convergence after abandonment should be tested using longitudinal monitoring. (3) Environmental heterogeneity: within-treatment environmental heterogeneity was not explicitly quantified, so the increase in homogeneous selection should not be interpreted as direct evidence of measured environmental homogenization. (4) Indirect functional inference: PICRUSt2 predictions and ITS guild annotations cannot substitute for functional gene-level, transcript-level, or process-rate measurements. (5) Replicate number: *n* = 3 plots per treatment limits statistical power, and the high bacterial PERMANOVA *R*^2^ should be interpreted with awareness of this constraint.

Future research should move beyond this local cross-sectional design by establishing paired managed and abandoned plots within the same hillslope or watershed, increasing plot-level replication, and conducting repeated sampling through time. Such designs would allow management effects to be separated more clearly from spatial background variation and would test whether the inferred shift toward bacterial homogeneous selection represents a stable successional trajectory. Future work should also quantify within-treatment environmental heterogeneity, litter inputs, root traits, rhizosphere processes, and microclimatic conditions to identify the microhabitat filters that were not captured here. Finally, combining amplicon sequencing with metagenomics, metatranscriptomics, enzyme kinetics, and direct measurements of decomposition and nutrient-cycling rates would help determine whether the observed community assembly shifts translate into functional changes in bamboo forest soils.

## 5 Conclusions

In moso bamboo forests of Taohuajiang State Forest Farm in the central Hunan hilly region, management cessation and subsequent abandonment were associated with systematic soil environmental reorganization—particularly along gradients of total *K*, pH, and ammonium nitrogen—and with divergent restructuring of bacterial and fungal communities. Bacterial communities exhibited stronger and more deterministic responses associated with abandonment duration: Shannon diversity declined monotonically with abandonment, and assembly processes shifted progressively from dispersal limitation toward homogeneous selection. This transition may reflect stronger environmental filtering and successional convergence after management cessation, although direct evidence for reduced within-treatment environmental heterogeneity was not assessed. Fungal communities, while also undergoing compositional change, showed greater depth-dependence, more stochastic assembly, and weaker environmental predictability. Representative taxa showed directional shifts along core environmental gradients, with partial robustness across multiple statistical frameworks; predicted functional-potential patterns were partly consistent with community turnover but remain indirect inferences. This study provides a simultaneous comparison of bacterial and fungal assembly processes across soil depths and abandonment duration in moso bamboo forests of the central Hunan hilly region, extending the concept of divergent bacterial-fungal assembly to bamboo forest abandonment ecology. From a management perspective, the lower bacterial Shannon diversity observed under longer abandonment suggests that long-term unmanaged bamboo forests may be associated with reduced bacterial diversity, with potential consequences for the soil functions these communities underpin. These results suggest that management decisions regarding bamboo forest abandonment should consider soil bacterial community diversity as one component of belowground ecological integrity.

## Data Availability

The original contributions presented in the study are included in the article/Supplementary material, further inquiries can be directed to the corresponding author.
