# Supplementary material for: Management cessation drives divergent bacterial and fungal community assembly in moso bamboo forests of the central Hunan hilly region, China
Source: Front Microbiol. 2026 Aug 31;17:1880979. doi: 10.3389/fmicb.2026.1880979 (PMC13572612; doi:10.3389/fmicb.2026.1880979)
Supplement: Supplementary file 1 [file Table_1.docx]

# Supplementary Materials

## S1. Supplementary Methods

### S1.1. Functional prediction of bacterial communities (16S)

Functional profiles of bacterial communities were inferred using PICRUSt2 v2.5.1 following the recommended workflow: sequence placement, hidden-state prediction, gene family abundance inference, MetaCyc pathway reconstruction, and functional annotation mapping. Final outputs comprised enzyme commission (EC) number tables and MetaCyc pathway abundance profiles. Only functional categories relevant to carbon decomposition (e.g., beta-glucosidase, cellulose synthase), nitrogen cycling (e.g., nitrate and nitrite reductases, urease), and phosphorus transformation (e.g., phosphatase-related enzymes) were retained. Broad central metabolic pathways were excluded.

### S1.2. Functional annotation of fungal communities (ITS)

Fungal ecological functions were annotated using FUNGuild and FungalTraits. Only saprotrophic guilds (litter, wood, and unspecified saprotrophs) were retained, focusing on decomposition-related ecological functions.

### S1.3. Statistical analysis of functional data

Functional responses were analyzed using LMMs (response ∼ Treatment × Depth + (1 | Plot)). Bacterial functional profile abundances were log1p-transformed; fungal functional annotations were analyzed as relative abundances. Spearman correlations with the six core environmental variables were BH-corrected.

### S1.4. Limitations of functional inferences

Functional profiles inferred from 16S data are phylogenetic predictions. ITS-based results represent ecological guild classifications rather than direct measurements of functional gene abundance or activity, and should be interpreted as predicted functional potential only.

## S2. Supplementary Results

### S2.1. Functional shifts in bacterial communities

PICRUSt2-inferred functional potential showed management-associated changes in carbon, nitrogen, and phosphorus cycling functions. Carbon functions included 6-phospho-β-glucosidase and cellulose synthase; nitrogen functions included nitrite reductase, nitrate reductase, and urease; phosphorus functions involved phosphatase-related enzymes. Several carbon, nitrogen, and phosphorus cycling functions showed higher predicted abundance under AM20, while urease was relatively more abundant under EM, suggesting a shift in the structure of functional potential rather than a uniform directional change.

### S2.2. Functional shifts in fungal saprotrophic guilds

AM10 showed relative enrichment of litter-associated saprotrophs; AM20 was characterized by a broader representation of saprotrophic traits; EM generally exhibited lower relative abundances of several saprotrophic groups. These patterns reflect potential differences in substrate availability and decomposition-related ecological niches across management regimes.

### S2.3. Associations between functional traits and environmental variables

Urease activity correlated positively with cellulase activity; several carbon-degrading enzymes correlated negatively with total K and pH; nitrogen cycling functions also correlated negatively with total K. Fungal saprotrophic traits showed comparable patterns, with most groups negatively correlated with pH and total K. These associations are consistent with the interpretation that environmental variation is linked to shifts in both community composition and inferred functional potential.

## S3. Supplementary Figures and Table

**Fig. S1.** Phylum-level community composition across soil depths under different management treatments.

**Fig. S2.** Key microbial taxon responses across soil depths.

**Fig. S3.** Predicted bacterial functional profiles (PICRUSt2-based) across treatments (Z-score normalized).

**Fig. S4.** Correlations between microbial functional traits and soil environmental variables.

**Fig. S5.** Bacterial community responses along environmental gradients.

**Fig. S6.** Fungal community responses along environmental gradients.

**Fig. S7.** Composition of fungal saprotrophic guilds based on ITS amplicon sequencing.

TableS1_LMM_fixed_effects

| assay | response | response_label | effect_label | statistic | p_value | p_adj_bh |
| --- | --- | --- | --- | --- | --- | --- |
| environment | total_k_mg_g | Total K | Treatment | 637.203054067367 | 0.001 | 0.000 |
| environment | total_k_mg_g | Total K | Depth | 1.55077309046082 | 0.239 | 0.250 |
| environment | total_k_mg_g | Total K | Treatment x Depth | 2.20986184934365 | 0.109 | 0.119 |
| environment | ph | pH | Treatment | 330.185698069043 | 0.001 | 0.001 |
| environment | ph | pH | Depth | 152.144513983431 | 0.001 | 0.001 |
| environment | ph | pH | Treatment x Depth | 21.6787078524411 | 0.001 | 0.001 |
| environment | organic_matter_g_kg | Organic matter | Treatment | 48.0845325311783 | 0.001 | 0.001 |
| environment | organic_matter_g_kg | Organic matter | Depth | 277.173185405626 | 0.001 | 0.000 |
| environment | organic_matter_g_kg | Organic matter | Treatment x Depth | 27.2540446127828 | 0.001 | 0.001 |
| environment | ammonium_n_mg_kg_fresh | Ammonium N | Treatment | 22.6561897627672 | 0.000 | 0.000 |
| environment | ammonium_n_mg_kg_fresh | Ammonium N | Depth | 75.2303519318751 | 0.000 | 0.000 |
| environment | ammonium_n_mg_kg_fresh | Ammonium N | Treatment x Depth | 5.14276916417159 | 0.006 | 0.009 |
| environment | cellulase_activity | Cellulase activity | Treatment | 65.8574923439546 | 0.001 | 0.001 |
| environment | cellulase_activity | Cellulase activity | Depth | 136.389921868823 | 0.001 | 0.001 |
| environment | cellulase_activity | Cellulase activity | Treatment x Depth | 22.3508553293293 | 0.001 | 0.001 |
| environment | polyphenol_oxidase_activity | Polyphenol oxidase activity | Treatment | 23.9569224628826 | 0.001 | 0.001 |
| environment | polyphenol_oxidase_activity | Polyphenol oxidase activity | Depth | 0.248718628267487 | 0.782 | 0.782 |
| environment | polyphenol_oxidase_activity | Polyphenol oxidase activity | Treatment x Depth | 2.26950257545986 | 0.102 | 0.116 |
| 16S | shannon | Shannon | Treatment | 30.8841646629551 | 0.000 | 0.000 |
| 16S | shannon | Shannon | Depth | 3.33944858635416 | 0.058 | 0.074 |
| 16S | shannon | Shannon | Treatment x Depth | 2.78182204918079 | 0.058 | 0.074 |
| ITS | shannon | Shannon | Treatment | 4.37865596126215 | 0.023 | 0.032 |
| ITS | shannon | Shannon | Depth | 2.82834280076394 | 0.077 | 0.092 |
| ITS | shannon | Shannon | Treatment x Depth | 8.80040710420661 | 0.000 | 0.000 |
